# A Unimodal Species Response Model Relating Traits to Environment with Application to Phytoplankton Communities

**DOI:** 10.1371/journal.pone.0097583

**Published:** 2014-05-16

**Authors:** Tahira Jamil, Carla Kruk, Cajo J. F. ter Braak

**Affiliations:** 1 Biometris, Wageningen University and Research Centre, Wageningen, the Netherlands; 2 Department of Mathematics, COMSATS Institute of Information Technology, Islamabad, Pakistan; 3 Laboratory of Ethology, Ecology and Evolution, IIBCE Functional Ecology of Aquatic Systems, Limnology, Facultad deCiencias, Montevideo, Uruguay; University of Oxford, United Kingdom

## Abstract

In this paper we attempt to explain observed niche differences among species (i.e. differences in their distribution along environmental gradients) by differences in trait values (e.g. volume) in phytoplankton communities. For this, we propose the trait-modulated Gaussian logistic model in which the niche parameters (optimum, tolerance and maximum) are made linearly dependent on species traits. The model is fitted to data in the Bayesian framework using OpenBUGS (Bayesian inference Using Gibbs Sampling) to identify according to which environmental variables there is niche differentiation among species and traits. We illustrate the method with phytoplankton community data of 203 lakes located within four climate zones and associated measurements on 11 environmental variables and six morphological species traits of 60 species. Temperature and chlorophyll-a (with opposite signs) described well the niche structure of all species. Results showed that about 25% of the variance in the niche centres with respect to chlorophyll-a were accounted for by traits, whereas niche width and maximum could not be predicted by traits. Volume, mucilage, flagella and siliceous exoskeleton are found to be the most important traits to explain the niche centres. Species were clustered in two groups with different niches structures, group 1 high temperature-low chlorophyll-a species and group 2 low temperature-high chlorophyll-a species. Compared to group 2, species in group 1 had larger volume but lower surface area, had more often flagella but neither mucilage nor siliceous exoskeleton. These results might help in understanding the effect of environmental changes on phytoplankton community. The proposed method, therefore, can also apply to other aquatic or terrestrial communities for which individual traits and environmental conditioning factors are available.

## Introduction

All organisms have preferred environmental conditions in which they can survive, grow and reproduce optimally. Each species is, therefore, largely confined to a specific interval along an environmental variable. This concept can be extended from one environmental variable to many. Each species is, thus, presumed to occur in a characteristic, limited range of the multi-dimensional habitat space, called its ecological niche, and within this niche, each species tends to be the most abundant around a specific environmental optimum [1]. Therefore, the distribution of species along an environmental gradient is usually unimodal.

The simplest unimodal (non-negative) species response curve is the Gaussian response curve. It is symmetric and bell-shaped with three ecologically interpretable niche parameters [2], [3]: the optimum (centre of the niche), tolerance (width of the niche) and maximum value of the response. The model can be fitted by nonlinear regression, but it is easier to first reparametrize it as a generalized linear model (GLM) with a second order polynomial in the environmental variables and then fit it to data by any of the statistical packages that can handle GLMs [4], [5]. GLM can be fitted to presence-absence, counts or biomass data with appropriate link function.

The conceptual basis of matching species traits to environmental variables are credited to Southwood [6], [7], but started already with Tansley [8] and Pearsall [9] and was well-developed by Grime [10] for plants. Further improvement was done by Keddy [11] to predict community organization in an environment from a species pool and species traits. Important steps in this process are to construct species niches in environment space and to consider traits that directly or indirectly related to fitness and are easy to estimate for any species and organism [12], [13].

Phytoplankton is a diverse group of microscopic photosynthesizing algae and cyanobacteria. Small size (0.41 µm–1 mm), short generation times (0.5 to 2 d^−1^) and high abundances (10^7^ or more cells ml^−1^) make phytoplankton community dynamics discernible for a human observer and facilitate experimentation [14], [15]. Furthermore phytoplankton is fundamental for maintaining global biogeochemical cycles and trophic webs of pelagic ecosystems [16], and their excessive growth is one of the main concerning aquatic quality problems [17]. To understand what factors regulate their assembly and dynamics, it is necessary to comprehend how traits of species influence their response to the environment.

Following seminal works by Southwood [7] and Townsend and Hildrew [18], trait-based approaches have been increasingly applied to explain and predict response of phytoplankton species to environmental conditions. The main traits of phytoplankton species are the organisms' growth abilities, their form of resources acquisition (nutrients and light) and their capacity to evade loss processes (i.e. grazing, sedimentation). Different combinations of traits and environmental gradients have been used to define these axes [19]–[21]. Formalization of the approach has been done mainly by Reynolds identifying species preferences and tolerances [22], [23]. Other approaches cluster the species based on their functional traits and then summarize their response to environmental change [15], [16], [24]. These studies reveal that traits could offer new insights into phytoplankton ecology. Moreover, the inclusion of both continuous and categorical traits is fundamental to represent well species performance along environmental gradients [33].

A statistical approach was developed by Jamil et al. [25] to relate species traits to environment using an extension of GLM, namely the generalized linear mixed model (GLMM). It uses the environmental variables linearly and the regression parameters are made dependent on the species traits. By adding squared environmental variables to the model, it is able to fit niches, that is unimodal response to the environmental variables, but the downside of this approach is that the regression parameters of linear terms and the squared terms have no intuitive meaning and no ecological interpretation. By contrast, the optimum, the tolerance and the maximum of the Gaussian response model are interpretable parameters and we would like to model them in terms of the species traits. One could also consider a two-step approach that first derives estimates of the optimum, tolerance and maximum for each species separately by GLM and then regresses these in turn on to the species traits. Though two-step approaches could be contemplated, estimation errors can be reduced by the integrated approach proposed in this paper. It relates species traits to the environment via statistical models that explicitly acknowledge the concept of the ecological niche, i.e. models that are unimodal in terms of the environmental variables. Other approaches such as canonical correspondence analysis [26] and RLQ [27], [28] could handle unimodal data but without explicit models. These methods are very handy with unimodal data but are linear after transformation.

In this paper, we propose a Gaussian model [4] for binary data with linear trait submodels for the parameters. It models the occurrence probability of species in term of traits and environmental variables. We term it the trait-modulated Gaussian logistic model. It is hard to fit with available (generalized) nonlinear mixed model software. Instead, we take a Bayesian approach and fit the model using OpenBUGS (Bayesian inference Using Gibbs Sampling) [29]

The identification of traits responsible for explaining the variation in the response curve parameters is akin to the familiar model selection dilemma in regression. The challenge is to select a small subset of the trait variables that explain a large fraction of the variation in the response parameters. We use the Bayesian variable selection method of George and McCulloch [30] extended in Yuan and Lin [31] for trait selection. The same approach is applied to find the linear combination of environmental variables that best explains the species data through trait-modulated Gaussian logistic response curves.

The methods are illustrated using phytoplankton community data with corresponding, environmental variables and morphological traits. Morphological traits are related to species ecological performance [14], [15], [32] and are easy to estimate for any organism [33] and predictable from environmental variables [34]. Thus phytoplankton is an excellent model for combining differences among species in their distribution along environmental gradients and their differences in terms of morphological traits. The data set includes 60 species observed at 203 sites, 11 environmental variables and 6 morphological traits. This data set has shown a strong unimodal structure using generalized linear mixed models [35]. This is the first paper to attempt an explicit unimodal model for phytoplankton data.

In the present paper, we describe the trait-modulated Gaussian model, Bayesian variable selection and its implementation using MCMC algorithms in OpenBUGS. Then a case study on phytoplankton has been presented, showing how the Bayesian variable selection method selected the important environmental variables and traits. Finally, we compare the presented model with RLQ analysis, which is a popular ordination-based method to relate traits and environmental variables.

## Materials and Methods

### Unimodal response curve

In this section, we propose the trait-modulated Gaussian logistic model. The data we consider here is a *n × m* binary data table **Y** = [*y_ij_*] recording the presence (1)-absence (0) of *m* species (columns) in *n* sites (row), an environmental variable *x_i_* (*i* = 1,…, *n*) with quantitative measurements in the *n* sites, and an *m × K* data table **Z** = [*z_jk_*] of *K* quantitative or binary traits (columns) of the *m* species (rows), with *z_jk_* representing the value of the *k^th^* trait for the *j^th^* species. The subscripts *i*, *j* and *k* refer to site *i*, species *j*, and trait *k*, respectively. Later on we consider the case with multiple environmental variables; extension to count data is almost immediate and is detailed at the end of this section. We start with the Gaussian logistic model [4] with an extra random term for sites (Eq. 1). This term is added to account for the fact that species observed at the same site are likely to be correlated in occurrence, even after having taken account of the environmental (and trait) information [25], [35]. The model is phrased in terms of the logit of the probability of occurrence *p_ij_* = E(*y_ij_*), the expected value of the observation *y_ij_*, given the model, 
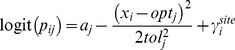
(1)with *x_i_* the quantitative known environmental variable, *opt_j_* the species optimum, *tol_j_* the species tolerance, *a_j_* a coefficient related to maximum probability of species *j*, and 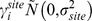, a normally distributed random site effect with variance 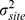. Recall that 
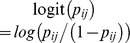
 with inverse 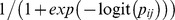. This model has thus a logistic form, and the model parameters *opt* and *tol* occur nonlinearly in the model function. The optimum on the gradient gives the location where the maximum probability of occurrence is attained and the tolerance gives the width of the response curve [4]. Given the occurrence probabilities {*p_ij_*} the data follow independent Bernoulli distributions, *y_ij_* ∼ Ber(*p_ij_*).

In the trait-modulated Gaussian logistic model, the parameters *opt*, *tol* and *a* are modulated by the *K* traits according to the linear sub-models
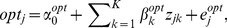
(2)

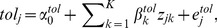
(3)

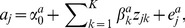
(4)with intercepts and slopes indicated by 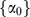 and 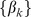 with a superscript for the corresponding parameter (subscript *k* indicates trait *k*). The error terms in these sub-models are 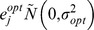, 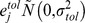 and 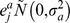 and are usually called random effects when inserted in Eq. 1. The resulting model is a nonlinear mixed model [36], where both fixed and random effects enter nonlinearly. We implement the model in OpenBUGS and fit it to phytoplankton community data. OpenBUGS uses Markov Chain Monte Carlo (MCMC), in particular Gibbs sampling, to generate a sample from the posterior distribution. For count data, we change in Eq. 1 the logit link function to log and the Bernoulli distribution for the data distribution to the Poisson or negative binomial distribution.

### Statistics for assessing contribution of traits variables

After fitting the model to data, the contribution of individual traits to the model can partly be assessed by the (standardized) size of their slope parameters 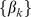 in Eqs. 2–4. In line with the usual definition of percentage variance explained in a model with multiple predictors, we measure the joint contribution of the *K* traits to the model for the optimum by [37], [38]

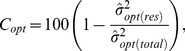
(5)where 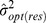 is the estimated variance in the model of Eqs. 1–4 and 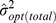 that in the model with all 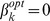, for *k* = 1,…, *K*. In Eq. 5 we compare the variance of the optimum in the model with and without traits [25]. Analogous definitions of percentage variance explained can made for the tolerance and the maximum. The variances are estimated by the posterior median.

It is worth pointing out that including traits in the model does not constrain the optimum (or tolerance or maximum), such as in constrained ordination [26]. The reason is that Eqs 2–4 include a random term, such as 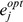, whereas such random term is not included in constrained ordination. We, therefore, do not expect much change in the variance explained on the level of the species data {*y_ij_*}. Inclusion of traits in our model attempts to shift unexplained variance, such as 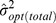, as much as possible to the fixed effects of a trait, thereby reducing the unexplained variance from 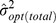 to 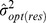.

### Bayesian variable selection

In data sets with many potential predictors, choosing an appropriate subset of traits and/or environmental variables is a challenging and important task. We use the Bayesian variable selection (BVS) approach of Yuan and Lin [31], the empirical Bayes estimator of which is closely related to the least absolute shrinkage and selection operator (LASSO) estimator [39]. The model analyzed here is unimodal response curve and parameters of the curve have a regression relation with a number of predictors. We apply variable selection to this regression relation within the full model, to obtain a parsimonious model with fewer variables. This model works best when most of the traits have no or only weak effects on the optimum, tolerance and maximum.

Bayesian variable selection can be influenced by choice of the prior. In principle there is considerable flexibility in the priors that could be used. Several Bayesian variable selection methods have been developed in recent years [30], [31], [40]–[42]. For details and for a review of Bayesian model selection methods see O'Hara and Sillanpää [43]. The naïve reader can think of these selection methods as advanced versions of “selection of variables” methods in regression, of which forward, backward and step-wise selection are the best known ones.

To keep the presentation simple, assume that the task is to explain an outcome 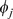 for species *j* (*j* = 1,…, *M*) using *K* trait variables with values *z_jk_*; *k* = 1,…, *K*. These variables may be continuous or discrete variables. The latter would be expanded to set of indicator variables [2]. Given a vector of regression parameters 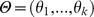, the response is modelled as a linear combination of the explanatory variables:
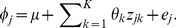
(6)


Here *µ* is the intercept and 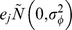 are the errors. The data are usually sufficiently informative to estimate the overall mean 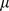 and the variance 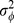 (the variation in response model parameter). Thus, we can use any reasonably noninformative prior distributions for these parameters. We used uniform priors for 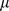 and 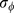, *i.e.* 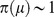 and 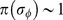.

For Bayesian model selection, we use a Slab and Spike prior [44] for 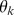. Slab and Spike priors offer useful model selection properties for the regression coefficients. With the spike it concentrates probability mass either exactly at or around zero, and with the slab it give a flat distribution elsewhere. Such a prior expresses the belief that there are coefficients close to zero and larger coefficients as well. To implement the model we adopt the hierarchical Bayes framework of Yuan and Lin [31] and assume a mixture prior for 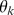




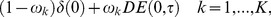
(7)where 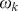 is a latent variable taking values 0 or 1, 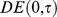 is the double exponential with density function 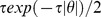 and 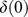 is the dirac function with point mass at 0. So if 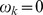, then 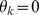, and otherwise it is double exponentially distributed with parameter 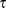. The double exponential is heavier tailed than the normal distribution and therefore can better accommodate large regression coefficients than with the commonly used normal prior 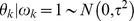 [31]. With the double exponential prior, the maximum a posteriori (MAP) estimator is the LASSO estimator [39], [45].

A typical choice for 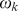 is Bernoulli with parameter 0.5. This prior assumes that the values 0 and 1 occur with equal probability. Note that in OpenBUGS normal distributions are defined in terms of a mean and precision, where precision  = 1/variance. The complete OpenBUGS model is given in the Appendix S1.

### Latent environmental variable

So far we have considered a single environmental variable denoted by *x_i_*. Community data are multivariate and several environmental factors have an effect on communities [46]. There are two ways to extend our model to multiple environmental variables. The first is to extend the quadratic form in Eq. 1 to a general quadratic form, 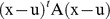 where **x** and **u** are now vectors with dimensions associated to the different environmental variables [47]. The second is to stay with Eq. 1 but to redefine *x_i_* as a linear combination of environmental variables, where then the challenge is to find the best linear combination given the data. The first approach uses far more parameters than the second and is more difficult to fit, and for those reasons we use the second approach in this paper. We extend this approach to find the best sparse combination by applying the same Bayesian variable selection approach to the environmental variables as we have described for traits in the previous section. The best sparse linear combination of (measured) environmental variables can be interpreted as a latent variable driving the phytoplankton communities.

For comparison, we also analysed the data by the fourth-corner method and RLQ. RLQ also yields latent variables (ordination axes). For details see [25].

### Initial values

We must supply starting values in order to estimate the parameters of a non-linear hierarchical model. Choosing appropriate values can be something of an art. OpenBUGS can crash when inappropriate values are specified.

For obtaining initial values for the Gaussian parameters *opt*, *tol* and *a* for a particular species consider the Gaussian logistic model, that is Eq. 1 without the random site effect,



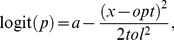
(8)where we drop the indices for sites and species for convenience. Instead of directly fitting this model to data of a particular species, we rewrite the model as the generalized linear model [4], [48] defined as a second-degree polynomial with logarithmic link function
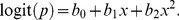
(9)


This model can be easily fitted as a generalized linear model (GLM) with logit link function and, if (estimated) 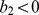, maximum likelihood estimates of the Gaussian parameters can be found by the following simple formulae [4], [48]:



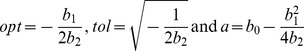
(10)


The coefficients 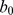, 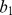 and 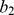 are thus easily transformed into coefficients representing the species optimum, tolerance and maximum probability. The point estimates of the Gaussian parameters thus obtained are identical to those obtained directly using nonlinear maximum-likelihood regression for the Gaussian logistic function. So GLM can be used to derive optimum and tolerance and probability of occurrence that will serve as starting values if 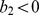. An optimum cannot be estimated well if it lies outside or near the edge of the environmental range, often leading to positive 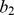. By augmenting the data with absences outside the environmental range, the optimum is well defined and lies within the newly created environmental range. We thus prevented any nonnegative 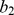 by augmenting the data with many zeros (absences) outside the observed range of the environmental variable. We thus viewed such cases as truncated unimodal curves, curves that would have been unimodal if the environmental range in the data were larger. The Bayesian data analysis was, of course, performed on the not-augmented data.

To estimate the initial values for 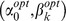, 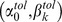 and 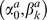 we regressed *opt*, *tol* and *a* on the traits mimicking Eqs 2–4.

### Deviance information criterion for model selection

For comparison of model quality, we use the Deviance Information Criterion (DIC; [49] defined as 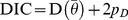
(11)where 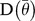
is the posterior deviance evaluated at the posterior mean of the parameter values and 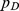 the estimated effective number of parameters in the posterior distribution. Spiegelhalter et al. [49] and OpenBUGS define 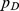 as the posterior mean of the deviance minus posterior deviance evaluated at the posterior mean of the parameter values,
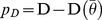
(12)so that 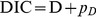
(13)


Sturtz et al [29] used this equation and approximated 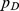
as half the posterior variance of the deviance, 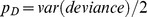, and estimated it by half the within chain variance of the deviance. We used this method for calculating DIC, as it is provided by the R2OpenBUGS function [29]. Eq. (11) shows that DIC can be viewed as the Bayesian counterpart of AIC model selection. The smaller the DIC value, the better the model.

The DIC statistic is in its early stages and is controversial [49]–[51]. Here we consider the DIC as a preliminary tool for comparing competing models. As with other model selection criteria, we caution that DIC is not intended for the identification of the best model, but rather merely indicates if a superior model exist within the given set of candidate models [52].

### Ethics Statement

The field studies were carried with all the permissions needed. Mainly in private land with permissions of the owners and in one national park with permission given by the DINAMA, Dirección Nacional de Medio Ambiente in Uruguay.

## Data and Statistical Analysis

Data is of 237 dominant species (at least 5% of total biomass in one lake) from 203 lakes located within four climate zones in South America, Europe, and North America, covering a wide range of environmental characteristics (Table 1).

**Table 1:** Range of environmental variables of lakes included in this study for the different regions: subpolar (49°04′–55°06′S), temperate (35°02′-39°08′S and 41°12′–52°36′N), subtropical (29°09′–34°09′S), and tropical (5°04′–23°05′S).

Latitude	Number of lakes	T (°C)	Ice cover	Z_mix_ (m)	Kd (m^−1^)	Cond (*µS cm* ^−1^)	TN*µg L* ^−*1*^)	TP*µg L* ^−*1*^)	TZ (org L^−1^)	Chl-a (*µeq L* ^−*1*^)
Subpolar*		13.4	yes	1.6	1.5	326	2106	438	2076	20.1
S	11	10.0–18.6		0.52–3.0	0.04–6.8	38–982	52–22993	15–9141	129–13855	<0.3–204
Temperate										
S	11	16.0	yes	1.4	1.0	671	1515	144	6151	31.9
N	107	0.4–33.0		0.13–7.0	0.01–4.0	53–4930	63–25913	<10–5600	105–26319	0.3–595
Subtropical		19.6	no	1.9	0.8	315	4286	388	1106	76.7
S	40	10.0–30.1		1.1–4.5	0.2–3.9	53–2095	68–37928	29–10087	3–13474	<0.3–284
Tropical		25.0	no	2.2	1.2	315	474	85.6	347	12.2
S	42	14.3–33.0		0.60–4.5	0.2–4.5	19–2100	35–1950	<10–394	12–2252	<0.3–178

Abbreviations as in Table 2. S: southern, and N: northern hemispheres.

*Sampling in subpolar lakes only included summer season.

For 107 lakes, information was obtained from published [53] and unpublished sources (a 1999 Dutch multi lake survey, G. van Geest and F. Roozen pers. comm. 2004). The remaining 104 lakes were sampled during 2005–2006 by standard procedures at least once during summer [54], [55]. For this study, we included only one summer sample per lake (203 cases). The sampling and sample-analyses protocols were comparable among the lakes. Lakes were sampled at random points covering the whole lake area. Water samples for nutrients and plankton were taken integrating the water column with a plastic tube (20 cm diameter) and combining from 3 to 20 random replicates in each lake. Phytoplankton samples were fixed in Lugol's solution. Zooplankton samples were filtered through a 50-µm sieve and preserved in a 4% formaldehyde solution. Environmental variables included temperature, inorganic suspended solids, water column mix depth, light attenuation coefficient, conductivity, alkalinity, total nitrogen, total phosphorus, total zooplankton abundance, cladocera abundance and chlorophyll-a. Details on sample analysis are provided in Kosten et al. [54] and Kruk et al. [55]. Chlorophyll-a is not an environmental variable *per se*, but it reflects a combination of processes related to the trophic state of the lakes, larger chlorophyll-a concentration reflect higher resources (nutrients, light) and community production (i.e. eutrophic state), while lower chlorophyll-a indicates lower nutrients and potentially lower production (i.e. oligotrophic state). Further, chlorophyll-a is usually applied as a measure of water quality and ecosystem trophic classification (i.e. oligotrophic to eutrophic states).

The volume of water and the method used to process phytoplankton data was selected to avoid “sampling size” effects applying rarefaction to plankton counts. After identifying all individuals to the species level, when possible, we counted until the number of species reached an asymptote, when no more new species appeared after 2–3 units of counting effort. This was done in random fields from fixed Lugol samples, using the settling technique (Utermöhl 1958). We examined the samples at multiple magnifications: organisms between 2 and 5 µm were counted at 1000X, those between 5 and 100 µm at 400X, and larger organisms at 200X. In this way the observed traits structure reflect well the species composition in the water. Furthermore, a higher total biomass it is not necessarily related to particular traits [33]. Information on trait assessment is given in [33], [34].

The environmental variables and traits variables are listed in Table 2, which also shows abbreviated names, the unit of measurement, number of missing values and whether the variable was transformed to natural logarithms in the analysis.

**Table 2:** List of environmental variables and trait variables with code and unit of measurement, number of missing values and indicator for the transformation to natural logarithms.

Variables	Code	Unit	Missing values	Log-transformation
**Environmental**				
Alkalinity	Alk	*µeq L* ^−1^	8	Yes
Chlorophyll-a	Chl-a	*µg L* ^−1^	13	Yes
Cladocera abundance	CLA	*org L* ^−1^	10	Yes
Conductivity	Cond	*µS cm* ^−1^	3	Yes
Inorganic suspended solids	ISS	*mg L* ^−1^	16	Yes
Light attenuation coefficient	Kd	*m* ^−1^	4	Yes
Temperature	Temp	°C	17	No
Total nitrogen	TN	*µg L* ^−1^	8	Yes
Total phosphorus	TP	*µg L* ^−1^	3	Yes
Total zooplankton abundance	TZ	*org L* ^−1^	8	Yes
Water column mix depth	Zmix	*m*	2	Yes
**Traits**				
Flagella (presence/absence)	Fla		0	No
Maximum linear dimension	MLD	*µm*	5	Yes
Mucilage (presence/absence)	Muc		0	No
Siliceous exoskeleton (presence/absence)	Si		0	No
Surface area	S/V	*µm* ^−1^	5	Yes
Volume	V	*µm* ^3^	5	Yes

We excluded species that occurred in less than 5% of the sites. The data set is of 203 sites and 60 species. We analysed the species data as presence/absence.

The data contained about 4% missing values in the trait and environment data (Table 2). Removing rows (species or sites) with missing values is an option but that means loss of information. Another option is to do imputation. Before imputation, those variables that were clearly not normally distributed were log-transformed to justify the assumption of normality in the imputation procedure (Table 2). Data Imputation was performed using the MICE R-package [56] using the method “mean” for continuous variables and method “logreg” for binary variables. Finally, each environmental variable and each trait variable was centred and scaled so that the sample mean is zero and the sample standard deviation is 1.

We fitted the Gaussian logistic model to the phytoplankton data with and without trait variables by OpenBugs. Each model was run for each environmental variable for 10,000 MCMC iterations, discarding the first halves as burn-in. For selecting the best sparse linear combination of environmental variables, we ran the Markov chain for 100,000 iterations and discarding the first halves to remove the dependence on the starting values and to allow adequate convergence. In this case, convergence of MCMC was very slow.

For all these analysis, the MCMC simulation were performed in the Bayesian software OpenBUGS, linked from the R statistical computing software [57] by R2OpenBUGS [29]. For each analysis, we ran three parallel chains with starting values estimated as explained above for some parameters; starting values for other parameters were randomly generated.

To give more insight in the importance of the traits for the niche structure, we divided the species into two groups (species with optimum less than zero and species with optimum greater than zero on the latent variable, not using traits) and tested for trait differences between the two groups of species (using Wilcoxon rank sum test for quantitative traits and chi-squared 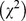 tests for binary traits).

## Results

**Figure 1:**
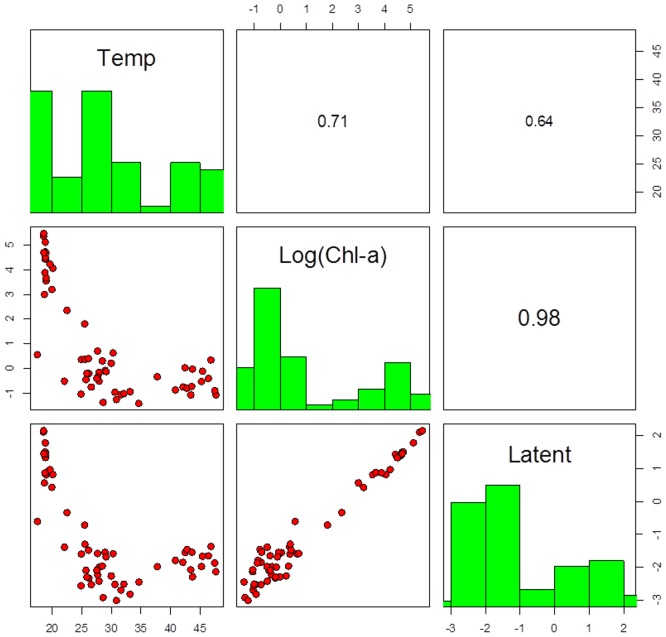
Pairplot of optima obtained from OpenBUGS output for temperature (in °C), chlorophyll-a (in log(*µg L* ^−1^)) and latent variable (arbitrary units).

**Figure 2:**
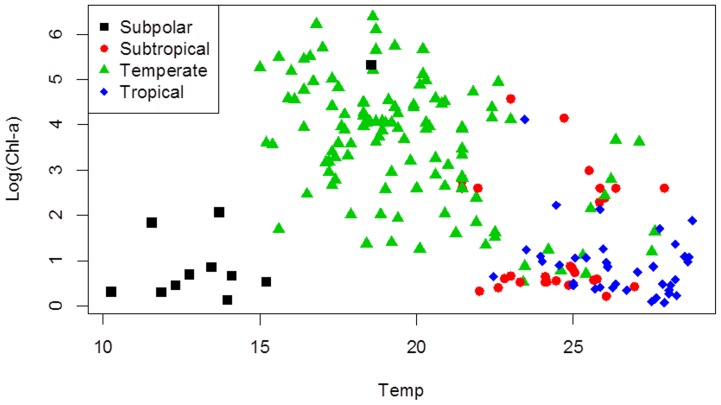
Bivariate scatter plot of environmental variables temperature (in °C), and chlorophyll-a (in log(*µg L* ^−1^)). Different climate zones are indicated with different colors.

As judged on the basis of the DIC in the Gaussian logistic models without traits, the best single environmental variable explaining the species niches was Chorophyll-a (Chl-a) (Table 3). The runner-up was temperature (Temp). Species and their parameters (*opt, tol, a*) values obtained from the OpenBUGS output for Temp and Chl-a are given in Table S1. The estimated optima regarding Chl-a and Temp gradients were negatively related (r = −0.71, Fig. 1) in agreement with the negative relation between Chl-a and Temp values in the lakes (r = −0.526, Fig. 2), particularly when excluding sub-polar lakes. By taking a combination of environmental variables, the model quality was further improved; this latent variable (Latent) yielded the lowest DIC (last line Table 3). DIC gave a similar rank order of environmental variables in the models with traits, with the latent variable being a clear winner (Table 3 last column). In terms of standardized variables (Table 2), the latent variable is defined as 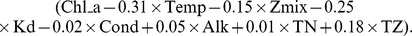


**Table 3:** Deviance information criterion (DIC) for individual environmental variables and the best linear combination of them in models with and without traits.

Env. variable	DIC (without traits)	DIC (traits)
Alk	6671^!^	7244^4^
Chl-a	6615	6609^1^
CLA	7738^!^	8531^10^
Cond	*	7133^3^
ISS	7736	7731^6^
Kd	8011	8011^8^
Temp	6842	6845^2^
TN	7816	7891^7^
TP	8418	8419^9^
TZ	7442	7442^5^
Zmix	8553	8552^11^
Latent	6283	6284^0^

The superscripts, rank of DIC in ascending order.

! negative *p_D_* value; * No convergence.

From the coefficients of the latent variable model, it emerged that the environmental variables Chl-a, Temp, Zmix, Kd and TZ are important, while ISS, TP and CLA are not.

The percentage of variance of the parameters (*opt, tol, a*) explained by the traits using Eq. 5 for the best three environmental variables (Temp, Chl-a and the latent variable) is displayed in Table 4. For Chl-a and the latent variable, the optimum could be much better explained by traits than the tolerance and maximum parameter. For temperature, the optimum and tolerance were about equally well explained. The models with traits and without traits gave similar DIC values (Table 3).

**Table 4:** Variance components in models without traits and with traits using the Yuan and Lin prior (Y) and Normal (N) prior for trait coefficients) and the fraction of variation explained.

	Variance component	Without traits	With traits (Y)	Fraction of variation (Y)	With traits (N)	Fraction of variation (N)
Temp	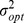	2.31	1.14	50.45	1.10	52.27
	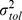	0.45	0.15	66.12	0.18	60.80
	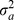	2.10	2.07	1.37	1.93	8.00
Chl-a	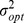	1.77	1.25	29.09	1.32	25.28
	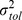	0.02	0.02	0	0.03	−28.00
	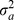	1.56	1.56	0	1.61	−3.39
Latent*	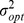	2.72	2.04	24.89		
	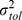	0.04	0.04	0.00		
	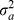	1.61	1.44	10.72		

^*^Latent variable, defined as the linear combination of standardized environmental variables.

**Figure 3:**
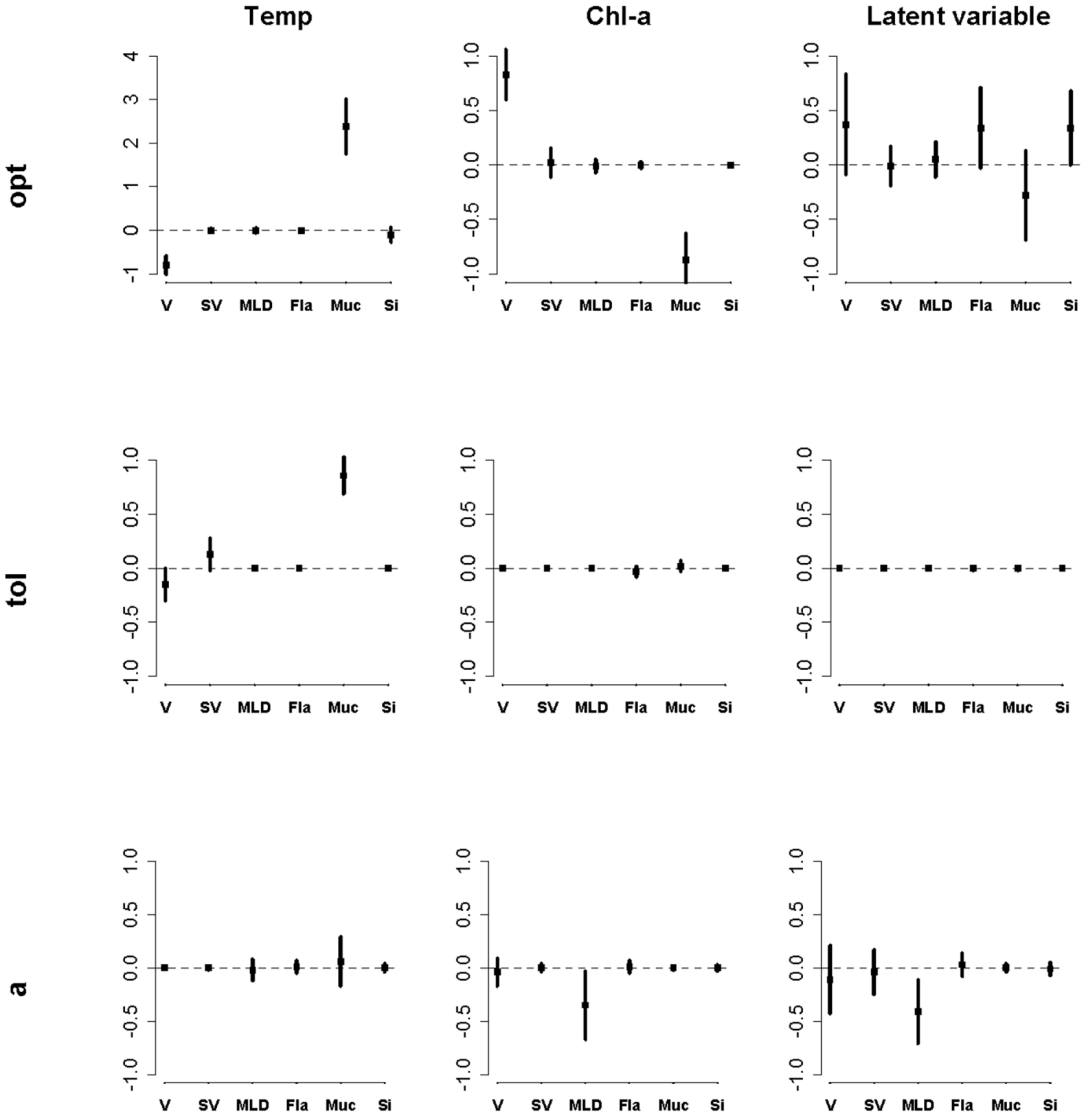
Coefficient estimate 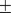 standard deviation for traits, when Gaussian response parameters (*opt, tol, a*) are regressed on traits for temperature, chlorophyll-a and the linear combination of environmental variables.

The regression coefficients with their standard deviations for the best three models are plotted in Fig.3. The traits mucilage, volume, flagella and siliceous structures were important for explaining the variation in optimum. Mucilage and maximum linear dimension (MLD) had non-zero coefficient for *a*, while Muc and S/V were related to tolerance for temperature. All trait coefficients for tolerance were zero for Chl-a and the Latent variable (Fig. 3, Table 4).

**Figure 4:**
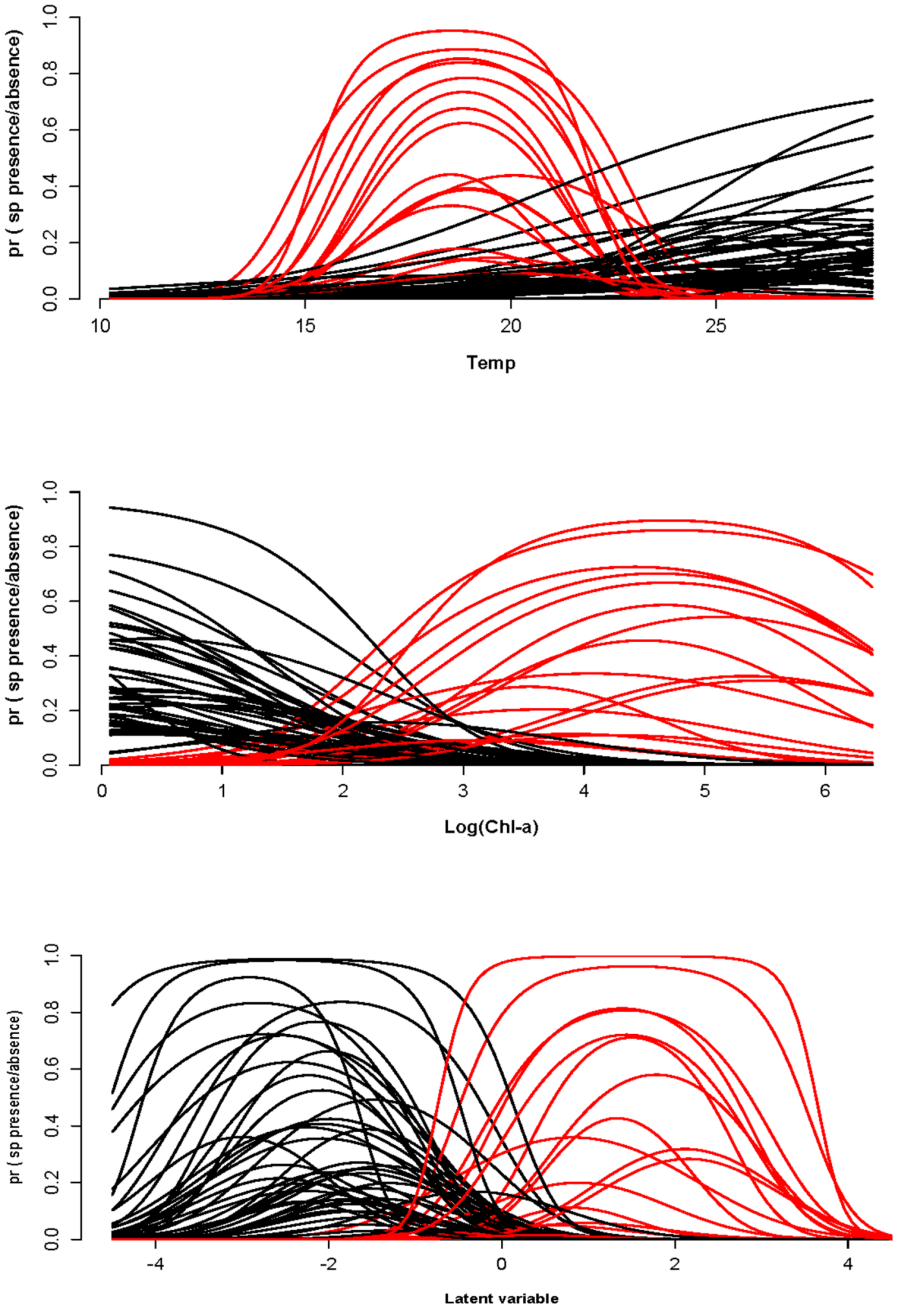
Response curves for species along the temperature gradient (in °C), Log(chlorophyll-a) (in log(*µg L* ^−1^)) and the latent variable.

Species response curves along the temperature gradient, log_10_Chl-a and the latent variable (Fig. 4) clustered into two groups of species. We classified the species in two groups on the basis of their optimum on the latent variable. The major components of the latent variable are Chl-a and Temp. Group 1 consists species with optimum less than zero and group 2 species with optimum greater than zero. Because of the definition of the latent variable, group 1 species have high temperature and low optimum in the Chl-a gradient while group 2 species have low temperature and high Chl-a. Low temperature species have well defined curve along temperature gradient while the optima of high temperature species are optimum lies outside the observed temperature range. The truncated response curve is nevertheless a part of Gaussian logit curve. For Chl-a gradient, group 2 species have large optima within the observed range while group 1 species have low optima outside the observed range. We tested for differences in morphological traits between the two species groups (Table 5, colors in Fig. 3). The Wilcoxon rank-sum test showed that V and S/V between two groups of species were significantly different (p<0.05). Flagella was absent in group 2 species and mostly present in group 1 species. Mucilage and siliceous was absent in Group 1 species. The presence/absence of these traits was significantly different (Table 5).

**Table 5:** Differences in (transformed) species traits among the two groups of species curves along the latent variable (Fig. 4c and Table S1), with significance for Wilcoxon rank sum test for quantitative traits and chi-square test for binary traits.

		Group 1	p-value	Group 2
**V**	mean	6.53	*	5.26
	sd#	3.11		2.23
**S/V**	mean	−0.23	*	2.5
	sd#	1.02		0.86
**MLD**	mean	2.61	n.s.	2.66
	sd#	1.18		1.26
**Fla** ^ &^	0	2	**	44
	1	14		0
**Muc** ^ &^	0	16	*	27
	1	0		17
**Si** ^ &^	0	16	**	35
	1	0		9

# sd  =  standard deviation;^ &^ Wilcoxon rank test; n.s. P>0.05; *P<0.05; **P<0.01.

**Figure 5:**
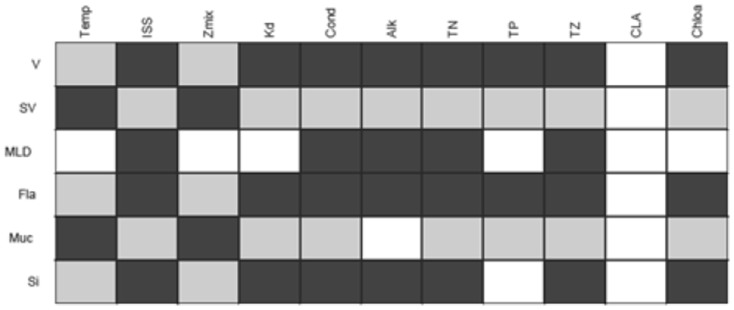
Result of pair-wise tests of trait-environment correlations using the fourth-corner method (non-white for significant (P<0.05) with light (dark) grey indicating negative (positive) relationships).

**Figure 6:**
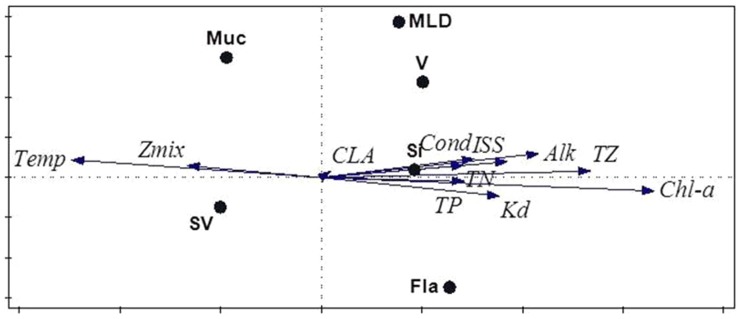
RLQ biplot of the Phytoplankton data. The first axis (horizontal) of the RLQ analysis explains 99% the variance in the fourth corner statistics, the second (vertical) 0.5%.

Nearly all trait-environment combinations appear significant as judged by the fourth-corner test (Fig. 5), except for environmental variable CLA. The first RLQ axis (Fig. 6) appears similar to the latent variable of the trait-modulated Gaussian model in that it is dominated by Chl-a and Temp with opposite signs. The signs of the coefficients of the remaining environmental variables also agree, except for Cond and Kd. Also, ISS and TP appear important in the RLQ, but have coefficient 0 in the latent variable. The CLA is unimportant in both analyses. The second axis explained very little variance. The traits in the left (right) hand side of Fig. 6 are negatively (positively) correlated with the first axis. The signs of these correlations can be compared with the trait coefficients for the optimum in the latent variable in Fig. 3 and agree for V, Fla and Si and Muc. The main difference is that SV and MLD stand out similarly in Fig. 6 as Muc and V, respectively, but are near zero in Fig. 3.

## Discussion

Many biotic and abiotic processes contribute to the variability in phytoplankton assemblages. The best model constructed here (the latent variable model) included temperature, resources (light and nutrients: Chl-a, Alk, Kd), as well as variables indicating loss processes (Zmix, TZ). These variables represent well the main mechanisms modulating phytoplankton including growing, resources gathering, and evasion of loss processes (hydrological washout, sedimentation and consumption by zooplankton) [19], [58]. The latent variable used to construct this model represented a gradient from lower to higher standing biomass (Chl-a, Alk) along with higher to lower temperature. This gradient showed decreasing light attenuation in the water column and shallower mixing zone of the water column. Total phosphorus did not have a significant coefficient, probably because its variability was represented well by chlorophyll-a concentration. Total zooplankton abundance also increases along this biomass gradient [59].

Chl-a was the most important individual variable describing the species niches. Chl-a is a measure of total phytoplankton biomass; it reflects the trophic state of the lakes, and therefore, resource availability (nutrients, light) [60]..In this sense, higher Chl-a is usually related to high nitrogen and phosphorus, and increased alkalinity, being associated to the effect of the watershed [61].

Temperature was the second most important individual variable describing the species niche. Temperature has important direct effects on phytoplankton metabolism and growth [58], [62]. Changes in water properties and water column mixing are also indirect effects of Temp that affected drastically phytoplankton community structure [63]. Chlorophyll-a and temperature are indicators of eutrophication and climate warming, and the focus of intensive current research. These processes dramatically influence aquatic ecosystems, thus modifying their communities and functioning, promoting species invasion and also modifying trophic interactions [38], [64]–[66].

An interesting result of our analysis is that, temperature and Chlorophyll-a gradients showed an opposite effect (Figs 1 and 2). Species with high temperature optimum did not increase their presence under high trophic states indicated by high Chl-a. Some authors have shown that the relative importance of temperature and nutrients change along the studied latitudinal gradients with higher effect of nutrients in temperature regions due to higher anthropogenic derived eutrophication [54], . Also it might be caused by differences in trophic interactions between warmer and cooler lakes [54], [67], [69], [70].

### How do different traits influence the species niche features (optimum, tolerance and maximum)?

Trait knowledge allows the prediction of the niche of a particular species and the comparison of species in terms of performances along environmental gradients [32], [53], [55], . Traits increased the environmental explained variance of species niches but not very much. One reason might be that the traits give the model more parameters but less freedom. But traits add the possibility to predict the response of a new species in a new environment.

Phytoplankton morphological traits reflect the ability to acquire resources (light and nutrients), to grow and to avoid mortality, through such processes as hydrological washout, sedimentation and consumption by grazers [19], [58]. Volume and S/V ratio influence specific growth rate, resource-uptake, and light-interception properties [22], [33], [75]. In general terms, smaller size and higher S/V potentiate higher growth rates and a greater tolerance to limiting light conditions [76]. Size also change sinking losses, and species responses to disturbance [58], [77].

Furthermore, grazing efficiency by filter-feeding zooplankton is influenced by phytoplankton morphology [14], [68], [78], [79]. The presence of mucilage provides controllable buoyant properties [80], may help maintaining an adequate microenvironment for cells and avoidance of grazing [81]. Also, survival may be prolonged by the facility of remaining as resting colonies in the sediment [82]. Mucilage does not contribute to biovolume in terms of photosynthetically active biomass whereas higher chlorophyll-a are related to higher biovolume of the phytoplankton community. Therefore increasing volume in terms of mucilage might be related to lower chlorophyll-a. In case of latent variable also the presence of flagella increased the optimum, flagella motility might allow algae to forage for nutrients and avoid grazing [83].

Finally, the presence of siliceous structures changes the location of the optimum increasing its position along the latent variable. The obligate presence of a siliceous wall increases cell density and organisms sink rapidly being excluded from illuminated waters depleted in assimilative sources of silica [77]. Furthermore, siliceous walls also have advantages against certain types of grazers [84] and viral infections [85] and the presence of siliceous spines might reduce losses because of grazing [86].

A general different consequence of the traits in the allocation of the optimum distribution was observed for temperature, as was also observed for the latent variable. Direct effect of temperature in organisms includes the acceleration of their metabolism, increasing their growth rates (higher C assimilation), their senescence rate (higher photo-respiration) and therefore decreasing their average size [87]. The negative effect of temperature in size was also observed in paleo-ecological studies [88], [89] and actual field analysis [63]. However, we found larger V and lower S/V at tropical temperatures, this was associated to the presence of mucilage that increases size without increasing cell size or numbers of cells per organism. The indirect effect is the consequence of temperatures in water properties, higher temperature causes stratification favouring smaller organisms that sink slowly would be favoured [63] in our case the benefited species were those with mucilage one of the most adequate ways of remaining in suspension [81].

We only included environmental variables associated to local environments in our analysis. The niche final structure would also depend on regional or global processes. However, the inclusion of functional traits as volume and shape is directly related to distribution processes and might correct for this limitation [90].

The two species groups had significant differences in trait composition. Low temperature species had an optimum of 17−18°C typical of temperate summers with wide trophic characteristics while high temperature species had an optimum value of 25°C and low trophic state conditions. High temperature-low trophic state species were composed of Chlorophyceae and Cyanobacteria, while low temperature species were represented of many phylogenetic groups. This is in accordance with recent literature showing that Chlorophyceae and Cyanobacteria are the most favored groups at higher temperatures [32], [53], [55], and in that these groups have large functional diversity.

A recent alternative explanation for the co-existence of many species is advocated by the combination of neutral theory of biodiversity and niche theory [91]. The theory of self-organized similarity (also referred to as ‘Emergent Neutrality’) proposes that there may be limited number of evolutionary self-organized functional groups of species (and corresponding niches), but that within each group an essentially unlimited number of ecologically equivalent species might co-exist neutrally [92]. Now-a-days new studies are recognizing this theory as potential explanation [93], [94] but still more research is needed.

#### Comparison with RLQ

RLQ ordination helped us to show that a single dimension (gradient) is sufficient for describing the trait-environment relationships (Fig. 6). In later stages of work we could extend our model to perform a similar test of dimensionality. But RLQ ordination has also its drawbacks. The RLQ ordination is simply an ordination of the fourth corner statistics [25]. RLQ neglects any existing inter-trait and inter-environment correlation. By contrast, our trait-modulated Gaussian model accounts for such correlations. As soon as a trait comes up in the model, any trait that is correlated needs to have greater or additional explanatory power to enter the model with a non-zero coefficient. The same applies to environmental variables. This is the likely reason that the environmental variables ISS and TP and the traits SV and MLD appear important in the RLQ (Fig. 6), but are not important for the latent variable and its optimum (Fig. 3), respectively. Moreover, RLQ treats the binary response data in an ad-hoc way, whereas our model is principled, namely, based on the binomial distribution. Our method builds a parsimonious multi-trait multi-environmental model in the sense of regression analysis. Our Bayesian shrinkage and selection approach to select a parsimonious model is the modern analog of the much-used step-wise regression approach. Because our method is model-based, one can predict responses in new situations (a new species with different trait values and or new lakes with different environmental values) and calculate uncertainty limits for these predictions. Therefore the model can be falsified when new data comes available.

## Conclusions

This paper presents a Bayesian approach to fit a unimodal species response model to phytoplankton community data, incorporating both environmental variables and species traits. Species response curves show that species are divided into clusters (Fig. 4) and variation within the cluster seems very low. DIC was useful to select the potentially important environmental variables. Temperature and chlorophyll-a (with opposite signs) describe well the niche structure of all species. In contrast to expectation, DIC did not show the importance of the traits in our models despite the fact that about 25% of the variance in the niche centres with respect to chlorophyll-a was accounted for by the traits (but in line with the fact that niche width and maximum could not be predicted). Volume, mucilage, flagella and siliceous structures are found to be the most important traits to explain niche differences in terms of optimum.

Of course, not all measurable features are equally important and some important features may perhaps be combined into a synthetic (latent) environmental gradient. It is formed by a linear combination of environmental variables that are presumed to considerably explain the species distribution. Volume, mucilage, flagella and siliceous structures were significantly different between two groups of species defined on the basis of their optimum with respect to the latent variable.

We assumed that species response on an environmental gradient has a symmetrical bell- shaped (Gaussian) curve. However, other types of response also occur quite common because interactions between species and extreme environmental stress may cause skewed or non-unimodal responses. The Bayesian approach can be extended to other parametric nonlinear models with parameters made dependent on traits.

Finally, not only in the case of phytoplankton but also for other communities, the identification of particular species groups favoured under particular scenarios might help interpret and forecast the effect of ecosystem anthropogenic modifications. For example, forecasting the vulnerable and favoured species, as well as their changes along latitudinal ranges and changing environments is a fundamental purpose that has to be as soon as possibly fulfilled.

## Supporting Information

Table S1 **Species names and parameters (** ***opt, tol, a*** **) values obtained from BUGS output for Temperature, Chlorophyll-a and Latent variable.** Pico: picoplankton, v: variety, ni: not identified.
(DOCX)

Appendix S1 **Bugs model for Latent variable (with traits).**
(DOCX)
