# Supplementary material for: A Unimodal Species Response Model Relating Traits to Environment with Application to Phytoplankton Communities
Source: PLoS One. 2014 May 16;9(5):e97583. doi: 10.1371/journal.pone.0097583 (PMC4023968; doi:10.1371/journal.pone.0097583)
Supplement: Table S1 — Species names and parameters ( opt, tol, a ) values obtained from BUGS output for Temperature, Chlorophyll-a and Latent variable. Pico: picoplankton, v: variety, ni: not identified. (DOCX) [file pone.0097583.s001.docx]

**Table S1.** Species names and parameters $\left( opt, tol, a \right)$ values obtained from BUGS output for Temperature, Chlorophyll-a and Latent variable. Pico: picoplankton, v: variety, ni: not identified.

|  |  |  | **Temperature** | | | **Chlorophyll-a** | | | **Latent variable** | | |
| --- | --- | --- | --- | --- | --- | --- | --- | --- | --- | --- | --- |
|  | **Species name** | **Gp** | **Opt** | **Tol** | **A** | **Opt** | **Tol** | **A** | **Opt** | **Tol** | **A** |
| 1 | *Aphanocapsadelicatissima* | 1 | 42.09 | 10.05 | 1.19 | -0.74 | 1.50 | 1.36 | -1.85 | 0.87 | 1.24 |
| 2 | *Aphanocapsaholsatica* | 1 | 40.82 | 9.91 | -1.19 | -0.86 | 1.32 | -0.66 | -1.79 | 0.75 | -1.00 |
| 3 | *Aphanocapsaincerta* | 1 | 46.39 | 11.51 | -0.21 | -0.40 | 1.46 | -0.67 | -1.65 | 0.86 | -0.73 |
| 4 | *Aphanotheceminutissima* | 1 | 37.76 | 9.70 | -0.68 | -0.33 | 1.38 | -0.24 | -1.98 | 0.98 | -0.29 |
| 5 | *Aulacoseiragranulata v granulata* | 2 | 20.10 | 2.45 | -0.25 | 4.06 | 1.59 | -0.68 | 0.82 | 1.15 | -0.77 |
| 6 | *Chlorella homosphaera* | 2 | 25.72 | 2.58 | -0.99 | -0.44 | 1.30 | -0.21 | -2.09 | 0.77 | 0.06 |
| 7 | *Chlorella minutissima* | 2 | 31.62 | 6.33 | -0.22 | -1.05 | 1.52 | 0.84 | -2.70 | 1.06 | 1.07 |
| 8 | *Chlorella vulgaris* | 2 | 27.92 | 5.25 | -0.77 | -0.50 | 1.50 | 0.16 | -2.42 | 1.03 | 0.54 |
| 9 | *Chlorococcales 4* | 2 | 26.62 | 3.83 | -2.24 | -0.74 | 1.36 | -1.11 | -2.53 | 0.85 | -0.74 |
| 10 | *Chroomonas sp.* | 2 | 18.99 | 1.90 | -0.46 | 3.55 | 0.91 | -0.91 | 0.81 | 0.67 | -0.93 |
| 11 | *Chrysococcus sp.* | 2 | 18.85 | 1.46 | 1.02 | 4.68 | 1.03 | 0.35 | 1.50 | 0.66 | 0.40 |
| 12 | *Cryptomonasbrasiliensis* | 2 | 27.94 | 4.59 | -0.93 | -0.15 | 1.10 | 0.06 | -2.00 | 0.71 | 0.34 |
| 13 | *Cryptomonasmarsonii v1.* | 2 | 27.70 | 4.66 | -1.21 | -0.34 | 1.19 | -0.16 | -2.23 | 0.78 | 0.19 |
| 14 | *Cryptomonasmarssonii v2.* | 2 | 26.21 | 3.04 | -2.35 | -0.20 | 1.12 | -1.44 | -2.33 | 0.69 | -0.89 |
| 15 | *Cryptomonas sp.* | 2 | 18.84 | 1.75 | 1.66 | 4.42 | 1.19 | 0.98 | 1.43 | 0.85 | 1.03 |
| 16 | *Cyanodictyonimperfectum* | 1 | 47.66 | 10.16 | 1.17 | -1.07 | 1.31 | 0.67 | -2.13 | 0.70 | 0.59 |
| 17 | *Cyclotella sp.* | 2 | 18.79 | 1.50 | 1.76 | 4.60 | 1.17 | 0.86 | 1.39 | 0.77 | 0.87 |
| 18 | *Cyclotellamengehiniana* | 2 | 24.93 | 1.93 | -1.26 | 0.37 | 1.49 | -1.23 | -1.60 | 0.91 | -0.98 |
| 19 | *Cylindrospermopsisraciborskii* | 2 | 30.30 | 3.16 | -1.58 | 0.64 | 1.25 | -1.93 | -1.59 | 0.74 | -1.58 |
| 20 | *Dinobryondivergens* | 2 | 22.08 | 5.37 | -2.95 | -0.51 | 1.15 | -1.50 | -1.39 | 0.75 | -1.92 |
| 21 | *Dyctiosphaeriumpulchellum* | 1 | 45.39 | 9.86 | -0.76 | -0.11 | 1.46 | -1.75 | -1.67 | 0.87 | -1.65 |
| 22 | *Epithemia sp.* | 2 | 18.69 | 2.36 | -1.82 | 3.00 | 1.32 | -2.32 | 0.57 | 0.86 | -2.16 |
| 23 | *Euglena sp.* | 2 | 18.87 | 1.58 | 0.51 | 4.49 | 1.05 | -0.17 | 1.33 | 0.65 | -0.12 |
| 24 | Eukaryioticnanoplanktonni | 2 | 18.81 | 1.83 | 2.06 | 4.73 | 1.19 | 1.82 | 1.51 | 0.77 | 1.93 |
| 25 | *Eutetramorusfotii* | 1 | 42.45 | 11.14 | -1.02 | 0.04 | 1.40 | -1.14 | -1.55 | 0.83 | -1.03 |
| 26 | *Gomphonema sp.* | 2 | 18.98 | 1.89 | -0.44 | 3.67 | 1.48 | -1.36 | 0.88 | 0.94 | -1.22 |
| 27 | *Gymnodiniumcnecoides* | 2 | 28.60 | 1.57 | -1.19 | -1.36 | 0.91 | 0.53 | -2.92 | 0.55 | 0.74 |
| 28 | *Gyrosigma sp.* | 2 | 18.78 | 1.88 | -1.52 | 3.89 | 1.10 | -2.05 | 0.87 | 0.59 | -1.88 |
| 29 | *Jaaginemagracile* | 2 | 17.51 | 5.48 | -2.42 | 0.56 | 1.43 | -1.86 | -0.61 | 0.35 | -0.78 |
| 30 | *Lemmermmaniellapallida* | 1 | 43.70 | 9.29 | -0.94 | -0.02 | 1.57 | -1.96 | -2.29 | 0.97 | -1.47 |
| 31 | *Lepocinclissalina* | 2 | 18.54 | 1.54 | -0.23 | 5.35 | 1.27 | -0.73 | 2.11 | 0.89 | -0.60 |
| 32 | *Merismopedia duplex* | 1 | 47.47 | 10.19 | -0.30 | -0.89 | 1.44 | -1.09 | -1.87 | 0.72 | -1.14 |
| 33 | *Merismopediatenuissima* | 1 | 46.81 | 11.45 | -0.08 | 0.35 | 1.52 | -1.00 | -1.37 | 0.91 | -0.87 |
| 34 | *Monoraphidiumcontornum* | 2 | 27.67 | 3.88 | -1.41 | 0.71 | 1.45 | -1.27 | -1.58 | 0.92 | -0.94 |
| 35 | *Monoraphidiumconvolutum* | 2 | 25.55 | 6.61 | -2.03 | 0.38 | 1.38 | -1.09 | -1.30 | 0.84 | -0.99 |
| 36 | *Oocystislacustris* | 1 | 43.42 | 12.25 | -0.87 | -1.06 | 1.58 | -0.32 | -2.08 | 1.08 | -0.69 |
| 37 | *Oocystismarsonii* | 1 | 42.79 | 12.29 | -1.58 | -0.79 | 1.51 | -1.08 | -1.46 | 0.91 | -1.48 |
| 38 | *Oocystisparva* | 1 | 43.59 | 11.85 | -1.91 | -0.72 | 1.45 | -1.62 | -1.54 | 0.88 | -1.90 |
| 39 | *Oocystis sp.* | 1 | 45.22 | 10.62 | -0.45 | -0.52 | 1.47 | -0.93 | -1.98 | 0.84 | -0.83 |
| 40 | *Peridinium sp.* | 2 | 19.64 | 2.14 | -1.76 | 4.22 | 1.26 | -2.09 | 0.97 | 0.87 | -2.12 |
| 41 | *Peridiniumumbonatum v umbonatum* | 2 | 25.86 | 3.14 | -2.38 | -0.19 | 1.37 | -1.67 | -2.30 | 1.00 | -1.40 |
| 42 | *Phacussp* | 2 | 18.84 | 1.52 | 0.74 | 5.11 | 1.23 | 0.17 | 1.79 | 0.80 | 0.21 |
| 43 | Pico chlorophyta 1 | 1 | 33.16 | 5.14 | 0.23 | -0.93 | 1.33 | 0.63 | -2.82 | 0.89 | 1.28 |
| 44 | Pico chlorophyta 2 | 2 | 30.61 | 4.66 | -0.69 | -0.95 | 1.33 | 0.23 | -2.52 | 0.79 | 0.59 |
| 45 | Pico cyanobacteria 1 | 1 | 34.64 | 7.60 | 1.17 | -1.40 | 1.36 | 3.40 | -2.45 | 0.86 | 3.26 |
| 46 | Pico cyanobacteria 2 | 2 | 24.88 | 3.93 | -2.52 | -1.03 | 1.43 | -1.24 | -2.57 | 0.88 | -1.00 |
| 47 | Pico cyanobacteria 3 | 2 | 32.10 | 4.22 | 0.92 | -1.01 | 1.28 | 1.25 | -2.51 | 0.70 | 2.00 |
| 48 | *Planktolyngbyalimnetica* | 2 | 29.97 | 4.46 | -0.99 | 0.22 | 1.45 | -0.95 | -2.26 | 1.02 | -0.44 |
| 49 | *Pseudanabena sp.* | 2 | 28.47 | 5.49 | -2.68 | 0.31 | 1.35 | -2.07 | -1.97 | 1.00 | -1.83 |
| 50 | *Raphidiopsismediterranea* | 2 | 25.49 | 3.80 | -2.43 | 1.81 | 1.32 | -2.14 | -0.72 | 0.73 | -1.77 |
| 51 | *Rhodomonasminuta* | 2 | 26.14 | 6.09 | -0.98 | 0.41 | 1.44 | -0.15 | -1.48 | 0.97 | -0.03 |
| 52 | *Scenedesmusellipticus* | 2 | 28.95 | 7.64 | -1.41 | -0.07 | 1.19 | -0.16 | -1.55 | 0.80 | -0.29 |
| 53 | *Strombomonas sp.* | 2 | 18.56 | 1.88 | -0.70 | 5.46 | 1.26 | -0.80 | 2.15 | 0.89 | -0.73 |
| 54 | *Synechococcusaquatilis* | 2 | 29.11 | 4.12 | -1.86 | -0.12 | 1.41 | -1.48 | -1.69 | 0.82 | -1.30 |
| 55 | *Synechococcusnidulans* | 2 | 27.53 | 5.22 | -1.38 | -0.39 | 1.58 | -0.56 | -2.09 | 1.03 | -0.40 |
| 56 | *Synedraacus* | 2 | 19.97 | 3.03 | -2.30 | 3.20 | 1.27 | -2.29 | 0.43 | 0.69 | -2.02 |
| 57 | *Tetraedron minimum* | 2 | 22.51 | 4.23 | -2.06 | 2.36 | 1.36 | -1.68 | -0.34 | 1.00 | -1.58 |
| 58 | *Tetraeëdroncaudatum* | 2 | 30.84 | 2.60 | -1.22 | -1.25 | 1.31 | -0.97 | -3.01 | 0.83 | -0.39 |
| 59 | *Trachelomonas sp.* | 2 | 18.94 | 1.65 | 1.30 | 4.68 | 1.17 | 0.70 | 1.44 | 0.81 | 0.62 |
| 60 | *Trachelomonasvolvocina* | 2 | 18.58 | 1.34 | 3.01 | 4.69 | 0.97 | 2.16 | 1.46 | 0.60 | 2.37 |
