# Supplementary material for: A Unimodal Species Response Model Relating Traits to Environment with Application to Phytoplankton Communities
Source: PLoS One. 2014 May 16;9(5):e97583. doi: 10.1371/journal.pone.0097583 (PMC4023968; doi:10.1371/journal.pone.0097583)
Supplement: Appendix S1 — Bugs model for Latent variable (with traits). (DOCX) [file pone.0097583.s002.docx]

**Appendix S1**

Bugs model for Latent variable (with traits)

########################################################################

# X0=Chl-a: X1=Temp; X2=ISS; X3=Zmix; X4=Kd; X5=Cond; X6=Alk;, X7=TN; X8=TP; X9=TZ; #X10=CLA

# Z= (V, S/V, MLD, Fla, Muc,Si)

# opt=Optimum; tol= Tolerance; logc= a

######################################################################################

model{ # N observations (species*sites)

for (i in 1:N){

y[i] ~ dbin(p.bound[i],1)

p.bound[i]<-max(0,min(1,p[i]))

logit(p[i])<-Xbeta[i]

Xbeta[i]<-logc[sp[i]]-0.5*pow((Xstar[i]-opt[sp[i]])/tol[sp[i]],2)+

b.site[site[i]]

Xstar[i]<-beta0*X0[i]+beta[1]*X1[i]+beta[2]*X2[i]+beta[3]*X3[i]+

beta[4]*X4[i]+beta[5]*X5[i]+beta[6]*X6[i]+beta[7]*X7[i]+

beta[8]*X8[i]+beta[9]*X9[i]+ beta[10]*X10[i]

}

for (j in 1:n.sp) {

opt[j] ~ dnorm(opt.hat[j],tau.opt)

opt.hat[j]<-b0+ inprod(b[],Z[j,])

tol[j] ~ dnorm(tol.hat[j],tau.tol)

tol.hat[j]<-c0+ inprod(c[],Z[j,])

logc[j] ~ dnorm(a.hat[j],tau.a)

a.hat[j]<-a0+inprod(a[],Z[j,])

}

for (j in 1:n.site) {

b.site[j] ~ dnorm(0,tau.site)

}

for ( k in 1:n.env){

beta[k]~ddexp (0,taubeta[k])

taubeta[k]<-1/varbeta[k]

varbeta[k]<-(1-gammbeta[k])*0.001+gammbeta[k]*10

gammbeta[k]~dbern(pi.beta) }

beta0<-1

a0 ~dnorm(0,.0001)

b0 ~dnorm(0,.0001)

c0 ~dnorm(0,.0001)

for ( k in 1:6){

a[k]~ddexp (0,taua[k])

taua[k]<-1/vara[k]

vara[k]<-(1-gamma[k])*0.001+gamma[k]*10

gamma[k]~dbern(pi.a)

b[k]~ddexp (0,taub[k])

taub[k]<-1/varb[k]

varb[k]<-(1-gammb[k])*0.001+gammb[k]*10

gammb[k]~dbern(pi.b)

c[k]~ddexp (0,tauc[k])

tauc[k]<-1/varc[k]

varc[k]<-(1-gammc[k])*0.001+gammc[k]*10

gammc[k]~dbern(pi.c)

}

tau.a<-pow(sigma.a,-2)

sigma.a~dunif(0,100)

tau.opt<-pow(sigma.opt,-2)

sigma.opt~dunif(0,100)

tau.tol<-pow(sigma.tol,-2)

sigma.tol~dunif(0,100)

tau.site<-pow(sigma.site,-2)

sigma.site~dunif(0,100)

pi.a<-0.5

pi.b<-0.5

pi.c<-0.5

pi.beta<-0.5

}
